# SNPs in GPCR Genes and Impaired Osteogenic Potency in Osteoporotic Patient Lines-Based Study

**DOI:** 10.3390/ijms252413594

**Published:** 2024-12-19

**Authors:** Julia Sopova, Olga Krasnova, Giomar Vasilieva, Anna Zhuk, Olga Lesnyak, Vitaliy Karelkin, Irina Neganova

**Affiliations:** 1 Institute of Cytology, Russian Academy of Sciences, St. Petersburg 194064, Russia; 2 Laboratory of Amyloid Biology, Saint-Petersburg State University, St. Petersburg 199034, Russia; 3 Institute of Applied Computer Science, ITMO University, St. Petersburg 197101, Russia; 4 Department of Family Medicine, North-Western State Medical University named after I.I. Mechnikov, St. Petersburg 195298, Russia; 5 Vreden National Medical Research Center of Traumatology and Orthopedics, St. Petersburg 195427, Russia

**Keywords:** osteoporosis, GPCR, next-generation sequencing, patient-specific stem cells, osteodifferentiation

## Abstract

G-protein-coupled receptors (GPCRs) have emerged as critical regulators of bone development and remodeling. In this study, we aimed to identify specific GPCR mutations in osteoporotic patients via next-generation sequencing (NGS). We performed NGS sequencing of six genomic DNA samples taken from osteoporotic patients and two genomic DNA samples from healthy donors. Next, we searched for single-nucleotide polymorphisms (SNPs) in GPCR genes that are associated with osteoporosis. For three osteoporotic patients and one healthy donor, bone biopsies were used to generate patient-specific mesenchymal stem cell (MSC) lines, and their ability to undergo osteodifferentiation was analyzed. We found that MSCs derived from osteoporotic patients have a different response to osteoinductive factors and impaired osteogenic differentiation using qPCR and histochemical staining assays. The NGS analysis revealed specific combinations of SNPs in GPCR genes in these patients, where SNPs in *ADRB2* (rs1042713), *GIPR* (rs1800437), *CNR2* (rs2501431, rs3003336), and *WLS* (rs3762371) were associated with impaired osteogenic differentiation capacity. By integrating NGS data with functional assessments of patient-specific cell lines, we linked GPCR mutations to impaired bone formation, providing a foundation for developing personalized therapeutic strategies. SNP analysis is recognized as a proactive approach to osteoporosis management, enabling earlier interventions and targeted preventive measures for individuals at risk. Furthermore, SNP analysis contributes to the development of robust, holistic risk prediction models that enhance the accuracy of risk assessments across the population. This integration of genetic data into public health strategies facilitates healthcare initiatives. This approach could guide treatment decisions tailored to the patient’s genetic profile and provide a foundation for developing personalized therapeutic strategies.

## 1. Introduction

Osteoporosis is a widespread age-related disease that affects over 200 million individuals globally, characterized by reduced bone mineral density (BMD), degradation of bone microarchitecture, and an elevated risk of fractures [1]. The rising prevalence of osteoporosis worldwide is accompanied by increasing healthcare costs [2]. Women over 50 years of age are particularly at risk, yet early diagnosis remains a challenge [3]. In the Russian Federation, the incidence of osteoporotic fractures mirrors global trends, with proximal femur fractures reaching 126,666 cases in 2015—a figure projected to rise by 70% by 2050 due to an aging population [4].

Current osteoporosis diagnostics primarily rely on patient medical history, physical examination, and BMD measurements, with BMD serving as the most reliable predictor of fracture risk [5]. Genetic factors significantly influence BMD, with estimates suggesting that 60–90% of low BMD cases are due to heritable factors. This has driven extensive research into the genetic underpinnings of osteoporosis and the discovery of potential predispositions. With advances in next-generation sequencing (NGS) technologies, studies now target specific genomic regions to identify gene variants associated with osteoporosis [6].

Osteoporosis is a complex, multifactorial disease influenced by both nonheritable and heritable risk factors. Among the nonheritable factors, age and estrogen levels play significant roles; while individuals of both genders can develop osteoporosis as they age, postmenopausal women are particularly vulnerable due to the decline in sex-steroid hormones, specifically estrogen. Additionally, lifestyle factors such as lack of physical activity, alcohol consumption, an imbalance in gut microbiota and smoking are often underestimated but have a profound impact on bone health and homeostasis [7,8]. On the genetic side, osteoporosis is associated with numerous heritable risk factors, including various genes that regulate bone metabolism, as well as their mutations and SNPs. In other words, osteoporosis is recognized as a polygenic disorder wherein the phenotype results from the cumulative effect of multiple gene polymorphisms. Genome-wide association studies (GWAS), leveraging the power of microarray and sequencing technologies, have identified numerous genes linked to osteoporosis, including those coding for extracellular matrix proteins (e.g., COL1A1/2) [9], cell receptors (e.g., VDR and LRP5) [10,11], and key components of signaling pathways such as WNT [12].

Among the genes implicated in bone health, G-protein-coupled receptors (GPCRs) have emerged as critical regulators of bone development and remodeling [13]. GPCRs are also known as seven-transmembrane domain receptors, which constitute the largest superfamily of receptors. This diverse family can be categorized into five primary groups: rhodopsin, adhesion, glutamate, frizzled/taste, and secretin. Members of these families are expressed in bone cells and play crucial roles in bone tissue homeostasis. In response to extracellular cues, GPCRs activate intracellular pathways through heterotrimeric G proteins. G proteins oscillate between inactive and active states; upon activation, the Gα (alpha) subunit dissociates from the Gβ (beta) γ (gamma) subunits. This dissociation triggers a cascade of intracellular events, amplifying GPCR signals via second messengers such as calcium ions (Ca^2+^) and cyclic adenosine monophosphate (cAMP). GPCRs are integral to bone remodeling, influencing both osteoblasts (bone-forming cells) and osteoclasts (bone-resorbing cells). A well-studied example is the parathyroid hormone receptor type 1 (PTH1R), which regulates osteoblastic lineage commitment by inhibiting peroxisome proliferator-activated receptor (PPAR), a key regulator of adipogenic differentiation [14]. Additionally, PTH1R mediates antiapoptotic signaling protein kinase A (PKA) in mature osteoblasts [14,15,16]. Another significant GPCR in bone metabolism is the calcium-sensing receptor (CaSR), which positively influences pro-survival and differentiation signaling pathways in osteoblasts and osteoclasts, respectively [17,18]. To date, disruptions or mutations in 26 different GPCRs have been associated with bone disorders, including osteoporosis [19,20]. As research progresses, new molecular targets within the GPCR family are being explored for their potential roles in osteoporosis diagnosis and treatment [21]. The therapeutic options for osteoporosis can be divided into two main types: antiresorptive therapy and anabolic therapy. The former aims to decrease osteoclastic differentiation and activity, thereby reducing bone resorption. This category includes several agents, with at least five key options: bisphosphonates; monoclonal antibodies targeting the Receptor Activator of Nuclear Factor Kappa-B Ligand (RANKL), such as Denosumab, which are considered as a first-line treatment for osteoporosis; estrogens; selective estrogen receptor modulators; and calcitonin [22]. On the other hand, anabolic therapies are designed to promote bone formation. Current osteoanabolic agents include antisclerostin monoclonal antibodies (e.g., romosozumab), parathyroid hormone receptor (PTH1R) agonists such as teriparatide, and strontium ranelate, which targets CaSR [23,24]. As observed, some of the most effective treatments for osteoporosis, including teriparatide, strontium ranelate, and calcitonin, primarily function by modulating the activity and functioning of GPCRs. While these GPCR-targeting drugs are already used in bone-related therapies, GPCRs remain underutilized in osteoporosis treatment despite their role in bone metabolism [16,25]. A deeper understanding of the molecular mechanisms governed by GPCRs in bone formation and resorption could pave the way for novel therapeutics.

Despite challenges in studying GPCR mutations—many of which are embryonically lethal, limiting the use of mouse models—patient-specific cell lines carrying specific GPCR mutations provide valuable tools for mechanistic and cellular investigations of these genes in health and disease. These cell lines also serve as promising platforms for new drug development and treatment screening, advancing personalized medicine in the management of bone diseases.

In this study, we aimed to identify patients with GPCR mutations from those diagnosed with osteoporosis and establish control groups without these mutations. From these patients, bone biopsies were used to generate patient-specific cell lines, which were analyzed for their osteogenic differentiation potential. By integrating NGS data with functional assessments of these cell lines, we linked specific GPCR mutations to impaired bone formation, providing a foundation for developing personalized therapeutic strategies. This approach could guide treatment decisions, such as prescribing antiresorptive drugs or bone-forming therapies tailored to the patient’s genetic profile.

## 2. Results

### 2.1. Osteoporotic Patients Have Multiple SNPs in GPCR Genes

**Figure 1:**
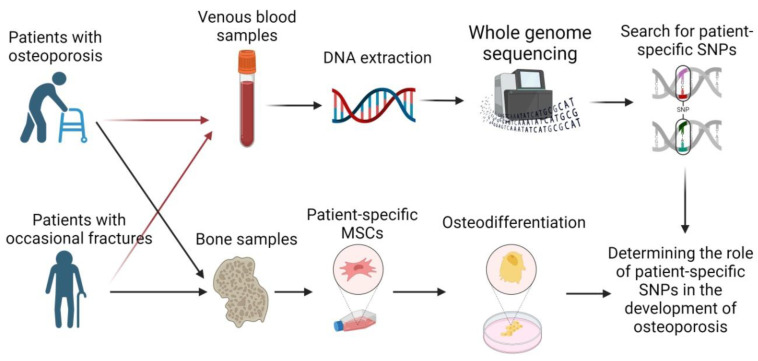
Combination of bioinformatics analysis and patient-specific cell lines for the determination of the patient-specific SNPs involved in the development of osteoporosis. Figure created with Biorender.

We used bone samples obtained from patients to collect patient-specific mesenchymal stem cells (MSCs) and venous blood samples to perform whole-genome sequencing according to the general schema of the experiment, presented in Figure 1.

Following sequencing, we analyzed the genetic data using a set of known pathogenic variants and genes associated with the development of osteoporosis to identify potential genetic contributors to the disease in our patient cohort. This set included 26 GPCR genes with specific single nucleotide polymorphisms (SNPs) linked to the progression of osteoporosis in humans, as detailed in [19].

**Figure 2:**
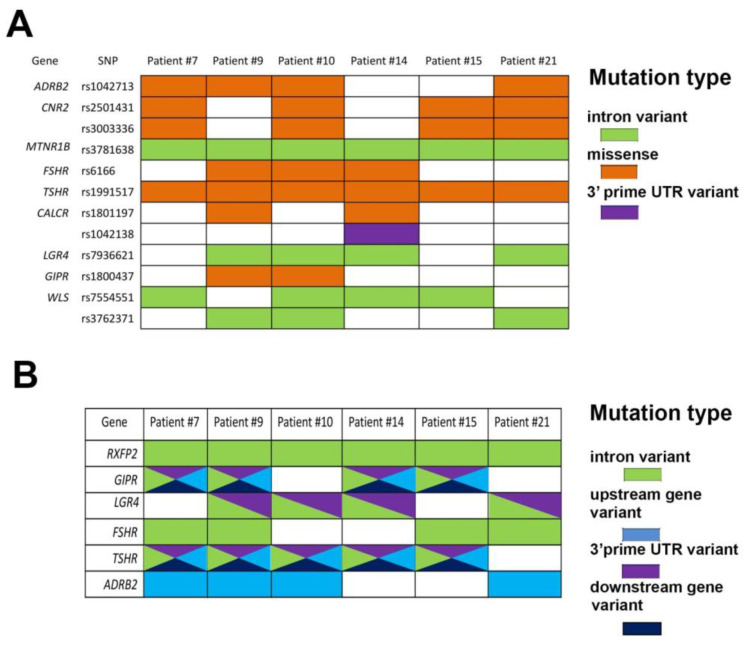
Osteoporotic patients have different sets of SNPs, potentially involved in osteoporosis progression. (**A**) The occurrence of known SNPs in GPCRs linked to BMD and osteoporosis progression in osteoporotic patients listed in this study. Missense mutations are shown in orange, intron variants in green, and 3′prime UTR variants in violet. (**B**) The distribution of SNPs in GPCRs found in four or more osteoporotic patients and absent in control patients. Intron variants shown in green, upstream gene variants in blue, 3′prime UTR variants in violet, and downstream gene variants in blue. Multicolored rectangles present different SNP types.

We found specific osteoporosis-linked SNPs in genes encoding key receptors involved in bone metabolism that are classified into rhodopsin (ADRB2, CNR2, MTNR1B, FSHR, TSHR, LGR4), secretin (CALCR, GIPR), and other T7M (WLS) receptor families (Figure 2A). Missense mutations in *ADRB2*, *CNR2*, *FSHR*, *TSHR*, *CALCR*, and *GIPR* genes were earlier associated with decreased BMD and osteoporosis in postmenopausal women [26,27,28,29,30,31] as leading to amino acid substitutions and the possible receptor structure and/or function change. In addition, we detected intron variants in *MTNR1B, LGR4*, and *WLS* genes that are associated with osteoporosis, increased fracture risk, and low BMD [32,33,34]. SNPs in introns and 3′ untranslated regions (rs1042138 in *CALCR*) might affect the expression of these genes, leading to the decreased/increased signaling.

Interestingly, we detected combinations of SNPs in noncoding regions of *RXFP2, GIPR*, *LGR4*, *FSHR*, *TSHR*, and *ADRB2* receptor genes, which were present in four or more osteoporotic patients and absent in control patients (Figure 2B). In *RXFP2* and *FSHR* genes, there were intron variants only; also, we found SNPs in the upstream sequence of the *ADRB2* gene. In *GIPR*, *LGR4*, and *TSHR* genes, we found multiple types of SNPs present either in all noncoding regions (*TSHR* and *GIPR*) or in intron and 3′prime UTR (*LGR4*). These SNP combinations were earlier not connected with any disease, and their impact on receptor expression is not clear.

For subsequent experiments, we chose three patients (#9, #10, and #14) due to their distinct SNP profiles. Data on patient #7 will be presented in a separate publication [35]. In addition to these patients, we also included one control individual (patient #8) for downstream phenotypic analyses.

### 2.2. MSCs Derived from Osteoporotic Patients Exhibit Impaired Osteogenic Differentiation Potential

**Figure 3:**
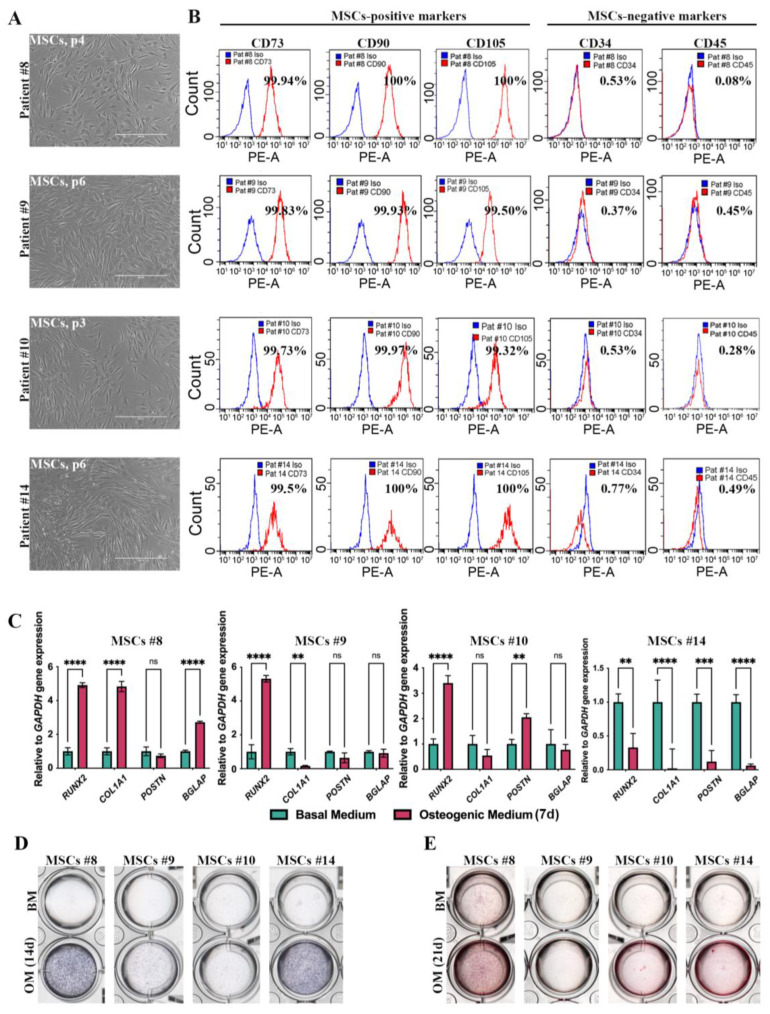
Characteristics of mesenchymal stem cells (MSCs) derived from patients’ bone samples. (**A**) Phase contrast representative images of MSCs derived from bone samples of #8 (control), #9, #10, and #14 patients. Scale bar 400 μm. (**B**) Immunophenotype of MSCs derived from bone samples of #8 (control), #9, #10, and #14 patients. Cells are positive for mesenchymal stem cell markers CD73, CD90, and CD105, while they are negative for CD34 and CD45. (**C**) *RUNX2*, *COL1A1*, *POSTN*, *BGLAP* gene expression in MSCs of #8, #9, #10, and #14 patients after 7 days of osteogenic differentiation. Data shown as mean SD, n = 3, with significant differences indicated with asterisks (ns—not significant, **—*p* < 0.01, ***—*p* < 0.001, ****—*p* < 0.0001). (**D**) Alkaline phosphatase activity in MSCs of #8 (control), #9, #10, and #14 patients after 14 days of osteogenic differentiation. Cells were grown on 24-well plate. (**E**) Alizarin Red staining of MSCs of #8, #9, #10, and #14 patients after 21 days of osteogenic differentiation. Cells were grown on 24-well plate. Abbreviations: BM—basal medium; OM—osteogenic medium.

For each osteoporotic patient and healthy individual, we successfully derived MSCs from bone samples, all of which exhibited a normal fibroblast-like phenotype when cultured in standard basal growth medium (Figure 3A). Flow cytometry analysis of surface markers (Figure 3B) showed that both control cells (patient #8) and cells from osteoporotic patients (#9, #10, and #14) expressed comparable levels of the main MSC-associated CD markers (CD73, CD90, and CD105), with no significant differences observed between the groups.

Subsequently, we sought to elucidate the impact of the genetic variants on the osteogenic differentiation potential. For this purpose, control and osteoporotic patient-derived MSCs were cultured in osteogenic differentiation medium (OD), and qRT-PCR analysis was performed on day 7 to assess the expression of key osteogenic markers. As shown in Figure 3C, the expression of the Runt-related transcription factor 2 (*RUNX2*) gene, encoding the main transcription factor of osteogenic differentiation, alpha-1 Type I Collagen (*COL1A1*), essential for bone formation, and *BGLAP* gene, which encodes osteocalcin—a vital protein for maintaining bone homeostasis—was elevated during osteodifferentiation of control patient’s #8 cell culture. The expression level of the Periostin (*POSTN*) gene, which encodes a key extracellular bone matrix protein, did not change significantly in the control patient’s cell culture during osteodifferentiation. All three osteoporotic patients’ cell cultures displayed an imbalance in osteogenic marker expression compared to the control. This imbalance was particularly pronounced in the cell culture of patient #14, where the expression of all osteogenic markers was significantly reduced. In the case of patient #9’s cell culture, we observed the increase only in *RUNX2* expression level; *COL1A1* had decreased expression level, and *BGLAP* and *POSTN* mRNA levels remained unchanged. During osteodifferentiation of patient #10’s cell culture, we detected the increase only in *RUNX2* and *POSTN* expression levels. The expression of *RUNX2* gene increased in both the control cells (patient #8) and cells from the osteoporotic patients #9 and #10 during osteogenic differentiation but decreased in cells from patient #14. Similarly, the expression of *COL1A1* gene was upregulated in the control cells (#8), while it was downregulated in cells from patients #9 and #14 and remained unchanged in cells from patient #10 during osteogenic differentiation. The mRNA level of the *POSTN* gene either decreased or remained unchanged in cells from patients #9 and #14, while it increased in cells from patient #10. The expression of *BGLAP* gene was significantly elevated in the control cells but remained either unchanged or significantly decreased in the osteoporotic patient cells, particularly in those from patient #14.

To further assess osteogenic differentiation potential, we performed alkaline phosphatase (ALP) staining after 14 days in OD. As presented in Figure 3D, the induced MSCs derived from patients #9 and #10 exhibited weak or negative ALP staining, contrasting sharply with the strong ALP staining observed in the control cells from patient #8. Unexpectedly, the induced MSCs from patient #14 showed almost no difference in ALP staining compared to control cells. These findings suggest that the efficiency of osteogenic differentiation varies significantly between control and osteoporotic patient-derived MSCs, as well as among the osteoporotic patients themselves. Moreover, the revealed disparity between qPCR analysis results and ALP staining for MSCs from patient #14 may be attributed to the patient’s comorbidities, specifically osteoporosis and type 2 diabetes mellitus (T2DM) (table in Section 4.1). The presence of these conditions can influence cellular behavior; for instance, insulin and other T2DM medications are known to enhance the proliferation and differentiation of osteoblasts [36]. This could potentially lead to premature commitment of the cells in culture, thereby affecting the observed results.

Finally, we analyzed the efficiency of calcium deposition, a key indicator of osteogenic differentiation and a hallmark of mature osteoblasts, in cells cultivated under standard growth medium and after 21 days of induction with osteogenic differentiation medium (OD) using Alizarin Red staining. As illustrated in Figure 3E, the calcium content varied between the control and osteoporotic patients’ groups under both culture condition. Cells from patients #9 and #10, who suffer from osteoporosis, exhibited the lowest ability to deposit calcium ions, a process vital for bone matrix mineralization in vivo. Importantly, the data obtained from the alkaline phosphatase (ALP) staining (Figure 3C) and Alizarin Red staining were consistent. Notably, in the control culture from patient #8, mature osteoblasts were present even before the addition of osteo-inductive factors. In contrast, cell cultures from patient #9 did not show positive staining with Alizarin Red, indicating the absence of mature osteoblasts in this patient’s culture. Interestingly, cultures from patients #10 and #14 demonstrated positive staining for Alizarin Red in OD, suggesting that a small pool of surviving osteoblasts might be maintained and further induced under these culture conditions.

## 3. Discussion

Throughout life, bone continuously renews and remodels through the coupled processes of bone resorption and formation, collectively known as “bone turnover”. In this process, old or damaged bone is eroded and replaced by new bone, ensuring the maintenance of strong bone tissue. Osteoporosis, a chronic skeletal disorder, is marked by low bone mass and reduced bone mineral density (BMD). This condition arises from disrupted bone microarchitecture, leading to an imbalance between bone formation and resorption, alongside abnormal matrix development. Recent studies have highlighted that genetic factors play a significant role in regulating the bone turnover process, with certain signaling pathways, such as those mediated by G-protein-coupled receptors (GPCRs), being particularly influential. GPCRs regulate key cellular processes in bone health, including osteoblast (bone formation) and osteoclast (bone resorption) activities. Disruption in GPCR signaling, due to mutations or dysregulation, can lead to imbalances in these processes, contributing to the development of osteoporosis.

In this study, through whole-genome sequencing analyses of a cohort of osteoporotic patients, we identified numerous single-nucleotide polymorphisms (SNPs) that had not been previously associated with osteoporosis in genome-wide association studies (GWAS), leaving their role in osteogenic differentiation largely unknown (e.g., intron variants of *RXFP2* gene were found in all osteoporotic patients but not in control individuals (Figure 2B)). Genetic factors influence bone health in a polygenic manner, with multiple gene variants, or single-nucleotide polymorphisms (SNPs), across various genes contributing to the risk of osteoporosis. The selected GPCRs listed in Figure 2 play a key role in cell signaling, with some being directly involved in signal transduction. The most impacted pathways include ERK1/2, p38, Akt1-FOXO, JNK, and WNT, which all are linked to cell differentiation, growth, and proliferation. Although mutations in these genes do not always directly influence BMD, several have been identified as pathogenic and linked to osteoporosis. Genetic factors influence bone health in a polygenic manner, with multiple gene variants, or single-nucleotide polymorphisms (SNPs), across various genes contributing to the risk of osteoporosis. However, these studies also highlight significant limitations in our current understanding of the cellular mechanisms, cell–cell interactions, and signaling networks essential for maintaining bone turnover without alterations.

Large-scale genome-wide association studies (GWAS) have illuminated the complexity of genetic networks that are important for bone metabolism, including osteoporosis [37,38]. Several large GWAS have investigated the genetics of osteoporosis, revealing BMD-associated genes, including *ESR1*, *LRP4*, *ITGA1*, *LRP5*, *SOST*, *SPP1*, *TNFRSF11A* (*RANK*), *TNFRSF11* (*RANKL*), and *TNFRSF11B* (*OPG*) [39]. A more recent study by Koromani and colleagues [40] demonstrated that the majority of susceptibility loci for fractures are implicated in BMD. Additionally, a comprehensive meta-analysis of osteoporosis, which included data from 17 GWAS encompassing 33,000 individuals of European and East Asian ancestry, confirmed the association of 24 pre-existing genetic loci and identified 32 additional loci associated with BMD; 14 of these loci were also linked to fracture risk [41]. Intriguingly, studies have shown that SNPs at multiple genomic loci contribute to bone strength and osteoporosis risk, though these variants are often very common in the general population and typically exert only a minor effect when considered individually [42].

**Figure 4:**
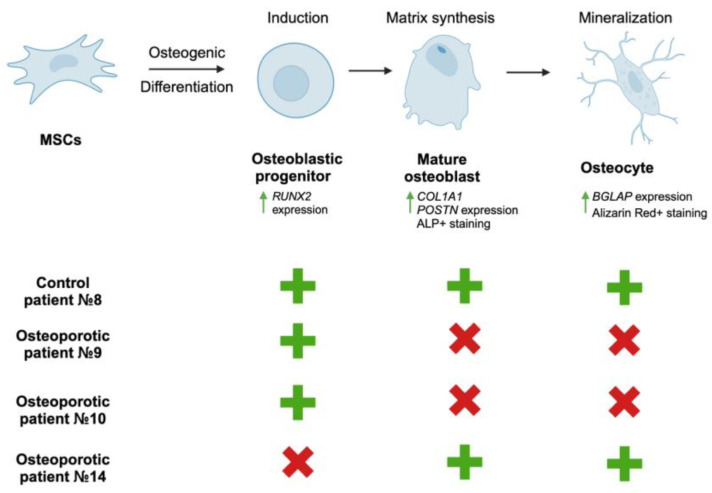
Simplified scheme summarizing osteogenic differentiation phases with schematic evaluation of osteogenic differentiation potency of cells derived from control and osteoporotic patients. Green arrows indicate increase in gene expression and specific staining, (+)—differentiation phase detected, (x)—differentiation phase absent.

In our study, we employed patient-specific cell cultures to link NGS data with the osteogenic competence of the cells, examined at all stages during induced osteoblast maturation in vitro (Figure 4). Analysis of cells derived from patient #9 indicated that a combination of missense mutations in the *ADRB2*, *FSHR*, *TSHR*, *CALCR*, and *GIPR* genes is associated with impaired osteogenic differentiation. qPCR analysis revealed an increase in *RUNX2* mRNA levels, suggesting the induction of osteogenic differentiation. However, the expression of genes involved in osteodifferentiation progression did not upregulate, as confirmed by histochemical staining (ALP staining, Alizarin Red staining), indicating ineffective osteogenic differentiation progression.

Cell culture from patient #10, carrying missense mutations in the *ADRB2*, *CNR2*, *FSHR*, *TSHR*, and *GIPR* genes, exhibited a moderate response to osteoinductive factors, as demonstrated by ALP and Alizarin Red staining. qPCR analysis showed increased expression of *RUNX2* and *POSTN* genes, indicating the cells’ ability to synthesize proteins important for further bone matrix development. Notably, this cell culture displayed positive ALP staining even before the addition of osteogenic differentiation media, suggesting the presence of osteoblasts in the culture prior to differentiation induction.

Of particular interest are the cells obtained from osteoporotic patient #14, which demonstrated the highest efficiency in response to the osteoinductive environment based on ALP and Alizarin Red staining for deposited calcium. However, gene expression analysis of these cells, which carry missense mutations in *FSHR*, *TSHR*, and *CALCR* genes, revealed decreased expression of all genes associated with osteodifferentiation. Osteogenic differentiation could be divided into at least three primary stages: initiation, matrix synthesis or maturation, and mineralization, and the transcriptional landscape varies significantly across these stages [43,44]. For instance, the *RUNX2* gene is upregulated during the initial phase of differentiation, but its mRNA levels decrease as osteogenesis progresses [45]. Conversely, the expression of the *BGLAP* gene, which encodes the protein osteocalcin crucial for bone matrix mineralization, shows an upward trend during later stages [45]. In the case of patient #14, whose comorbidity includes osteoporosis and T2DM (table in Section 4.1), the therapeutic drugs that he is taking for the management of diabetes may significantly affect MSCs derived from bone biopsies. Research has demonstrated that insulin and insulin-like growth factor 1 (IGF1) can directly influence both the proliferation and osteogenic differentiation of MSCs in vitro [46,47]. Thus, the observed disparity in gene expression and histological staining in MSCs of patient #14 may stem from the fact that we obtained bone biopsies immediately following surgery, so we obtained an already committed cell population with low osteogenic markers gene expression (Figure 3C). However, these cells were able to accumulate significant bone matrix components, as on the 14th and 21st days of OD, the cells exhibited positive staining for both ALP and Alizarin Red staining, respectively (Figure 3D,E).

Thus, at this stage, it can be inferred that osteoporosis in patient #9 is linked to decreased efficiency in bone formation. Conversely, patients #10 and #14 exhibit a moderate response to osteoinductive factors, potentially indicating that increased osteoresorption contributes to their osteoporosis. However, this assumption necessitates further study involving osteoclast cultures to assess resorptive characteristics in light of the present mutations.

Comorbidities (table in Section 4.1) have a profound impact on osteoporosis, often exacerbating the patient’s condition [48]. The prevalence of hypertension and other cardiovascular diseases (CVD) alongside osteoporosis increases with age, suggesting a significant overlap between these conditions [49,50,51]. A uniform factor in hypertension is elevated sympathetic tone, which primarily operates through the activation of adrenergic receptors (ARs), members of the GPCR superfamily [52]. β-adrenergic receptors (β-ARs) are expressed in almost all cell types, including bone cells [53]. Numerous studies have shown that excessive stimulation of β-ARs can lead to bone loss. However, patients with hypertension often take antihypertensive medications, including β-blockers, which have been found to have a protective effect on bone tissue integrity [53]. This highlights the complex interplay between these conditions and the potential benefits of specific pharmacological interventions in managing both hypertension and osteoporosis.

Chronic gastritis is notably prevalent among the elderly population. A key factor contributing to the progression of chronic gastritis is infection with Helicobacter pylori. Additionally, poor lifestyle, particularly smoking, can exacerbate the condition, negatively impacting both gastric health and overall bone health [54].

In our cohort, only one patient (patient #14) has been diagnosed with T2DM. This is intriguing, given the established connection between osteoporosis and diabetes, as highlighted in a meta-analysis of observational studies [55]. T2DM is known to be associated with osteoporosis, particularly in its later stages, where a reduced rate of bone formation is commonly observed [56]. Importantly, metformin, which is typically prescribed as a first-line treatment for T2DM, has been shown to positively influence osteoblastic activity while also having a negative impact on bone resorption [57].

Unlike conventional fracture risk assessment tools [58], identifying specific SNPs associated with osteoporosis represents a more proactive strategy in healthcare. This can facilitate early diagnosis and risk stratification. Patients with a genetic predisposition to impaired osteoblast maturation—which can lead to reduced bone formation—can be monitored more closely for signs of bone density loss and fractures. This monitoring allows for earlier interventions, potentially reducing osteoporosis-related morbidity and significantly improving patient outcomes. It is essential to recognize some limitations of this study. Firstly, increasing the sample size is crucial to ensure a more comprehensive representation of both male and female patients with diverse medical histories, which will enhance the accuracy of the results. Secondly, in order to shift from association description to revealing causal relationships, further in-depth investigations are necessary. Despite the limitations, this study provides valuable insights into the association between SNPs combinations and the progression of osteoporosis. These findings may pave the way for the development of robust patient-specific treatment strategies in the future.

## 4. Materials and Methods

### 4.1. Patients

The local Ethics Committee of the Vreden National Medical Research Center of Traumatology approved the study protocol (Saint-Petersburg, Russia) and adhered to the ethical principles outlined in the Declaration of Helsinki. All recruited patients have signed informed consent. Samples of the femur bone were harvested during surgery at the Vreden National Medical Research Center of Traumatology and Orthopedics. Two healthy donors with accidental femur fractures were used as control. Six patients with osteoporosis had osteoporotic fractures. Both healthy donors and female osteoporotic patients experienced menopause. All information about patients age, sex, and anamnesis is listed in Table 1.

### 4.2. NGS Sequencing

Genomic DNA was extracted from patient’s whole blood using a Blood & Cell Culture DNA Midi Kit (Quiagen, Venlo, The Netherlands). One μg of each DNA sample was used for whole-genome library preparation. DNA was sheared using an M220 Focused Ultrasonicator and microTUBE-50 tubes (Covaris, Woburn, MA, USA). The targeted library insert size was 350 bp. Genomic DNA libraries were constructed using TruSeq DNA PCR-Free Library Preparation Kits (Illumina, San Diego, CA, USA). The construction of genome libraries and all subsequent manipulations were carried out using supplemental reagent kits in accordance with the manufacturers’ protocols. The final libraries were quantified using the KAPA library quantification kit for Illumina sequencing platforms (KAPA Biosystems, Wilmington, MA, USA) and sequenced on the Illumina HiSeq 4000 platform (Illumina, San Diego, CA, USA).

### 4.3. Read Alignment and Variant Calling

FastQC (v0.12.0) was employed to evaluate the quality of the Illumina short reads [59]. Quality metrics were visualized using MultiQC (v1.23) [60]. The paired-end reads were aligned to the GRCh38 reference genome using the Burrows–Wheeler Aligner BWA-MEM (v0.7.17) with default parameters [61]. SAMtools (v1.20) was used to sort and index bam files. The BAM files were processed with Picard MarkDuplicates (v2.27.3) (http://broadinstitute.github.io/picard (accessed on 2 September 2024)) to identify and mark duplicates. Quality score recalibration was performed using GATK (v4.2.6.1) [62] through the BaseRecalibrator and ApplyBQSR steps, incorporating known sites from dbsnp155 (https://www.ncbi.nlm.nih.gov/snp/ (accessed on 2 September 2024)). The GATK HaplotypeCaller with GVCF parameter was used for variant calling, followed by merging the GVCF files using CombineGVCFs and conducting joint genotyping with GenotypeGVCFs. The variant call set underwent filtering based on Variant Quality Score Recalibration using VariantRecalibrator with known SNPs from dbsnp155 and ApplyVQSR. Additionally, variants were selected based on an allele depth (AD) greater than 5 and a read depth (DP) per sample exceeding 10, and only biallelic SNPs were included. Finally, variants were annotated using Ensembl Variant Effect Predictor (VEP) v111 [63]. Additionally, to explore SNPs associated with osteoporosis, we utilized the ClinVar database and GWAS Catalog. We focused on variants that are present in patients but absent in the control group for our analysis.

### 4.4. Cells Isolation and Determination of MSC Immunophenotype Using Flow Cytometry

MSCs were isolated from patients’ bone fragments according to protocol described in [64] and cultured under standard conditions. For immunophenotype FLOW cytometry assessment, cells after a single wash with PBS solution were resuspended in a solution of 0.25% Trypsin-EDTA, neutralized with growth medium with serum, and centrifuged. The cell pellet was then resuspended in 1 mL of bovine serum albumin (BSA, 0.5% (*w*/*v*) (Sigma, St. Louis, MO, USA)) in PBS to block nonspecific binding of antibodies for 30 min. Then the samples (2.5 × 10^5^ cells) were incubated on ice with fluorochrome-conjugated monoclonal antibodies at a concentration of 1 μg/mL in the dark. The following panels of monoclonal antibodies were used to determine positive (CD90/PE; CD73/PE; CD44/PE; CD105/PE; CD146/PE) and negative (CD34/PE; CD31/FITC; CD117/PE; CD45/PE) markers for immunophenotype of obtained MSCs. All antibodies were from BD Pharmingen (San Jose, CA, USA) except CD105/PE (eBioscience, San Diego, CA, USA). After 40 min of incubation with antibodies, the cells were washed twice with a solution of 0.3% BSA in PBS and analyzed using the LSR-II flow cytometer (BD Biosciences, San Diego, CA, USA) and Diva software package (v6.0). About 10,000 events were collected for each sample.

### 4.5. Osteogenic Differentiation of MSCs

Patient-derived MSCs were grown on 24-well plates (TPP, Trasadingen, Switzerland) for up to 80% of confluence. Then growth medium was replaced with osteogenic medium (DMEM with low glucose (1 g/mL D-glucose), 10% FBS, 100 U/mL Penicillin–Streptomycin, 10 mM β-glycerophosphate, 200 μM L–Ascorbic acid, and 100 nM dexamethasone (all Sigma Aldrich, St. Louis, MO, USA). Cells were refed every 3–4 days.

### 4.6. Analysis of Osteogenic Differentiation by Histochemical Methods

To stain for alkaline phosphatase (ALP), an early marker of osteoblasts, on day 14 of osteoinduction, cells were washed twice with PBS and fixed with 70% ethanol for 15 min at room temperature. After aspiration of ethanol, the cells were stained for 15–20 min with a solution containing BCIP/NBT (Merck Millipore, Burlington, MA, USA). Based on the formation of a purple-blue color, the reaction was stopped by washing in distilled water. The stained cells were photographed. After 21 days of osteogenic differentiation, MSCs were fixed with 70% ethanol for 1 h then stained with Alizarin Red (Service Bio, Wuhan, China) to reveal calcium deposits, a marker of mature osteoblasts. MSCs cultured in normal growth medium without osteoinducers were used as control cells.

### 4.7. Determination of Gene Expression and Osteoinduction Markers Using Real-Time PCR

Total RNA from cells cultured in osteoinduction and control growth medium was isolated on day 7 using the standard TRIzol protocol (Invitrogen, Oxford, UK). The concentration and quality of isolated RNA were assessed using a NanoPhotometer N60 spectrophotometer (Implen, Munich, Germany). Total RNA (1 μg) was reverse-transcribed using an MMLV RT kit (Evrogen, Moscow, Russia). Real-time PCR was carried out in an ABI 7500 thermal cycler (Applied Biosystems, Carlsbad, CA, USA) with the following temperature conditions: 10 min at 95 °C, 15 s at 95 °C, and 1 min at 60 °C for 40 cycles with cooling at the end to 4 °C. To carry out PCR, specific forward and reverse primers, listed in Table S1, were used. Data were analyzed using 7500 Software v2.0.6 and Ct values for each gene were obtained. Melting curve plotting was performed to ensure that the product consisted of a single amplicon. Threshold and baseline fluorescence values were set according to the manufacturer’s instructions (SABiosciences, Qiagen, Frederick, MD, USA). Data were analyzed using Qiagen software (http://www.sabiosciences.com/pcrarraydataanalysis.php (accessed on 1 September 2022)). Changes in gene expression levels (compared to control samples) were calculated using the ΔΔCt method. All experiments were performed in three independent replicates. Gene expression values are presented as mean ± standard deviation. Student’s *t*-test was used for pairwise comparisons between control and experimental samples. In statistical analysis, *p* < 0.05 was considered significant.

## 5. Conclusions

Altogether, our findings suggest that mutations in GPCR genes identified in patients with osteoporosis in our study are of great significance for osteogenesis and osteoblast maturation. Given that single-nucleotide polymorphisms (SNPs) contribute to individual differences in disease susceptibility, recent genome-wide association studies (GWAS) have significantly advanced our understanding of the genetic basis of osteoporosis, even when conducted in relatively small patient cohorts. Besides advancing our understanding of disease etiology, the identified and validated genetic markers are expected to aid in disease diagnosis and prediction. Functional follow-up studies in patient-specific cell cultures could potentially tailor treatment for each individual. For instance, the cell culture studies presented here suggest that osteoblastogenesis is impaired in patient #9 with rs1042713 in *ADRB2*, rs1800437 in *GIPR,* and rs3762371 in *WLS*, indicating that this patient requires therapy to support bone formation; patient #10 with moderate osteogenic differentiation capacity and rs1042713 in *ADRB2*, rs2501431 and rs3003336 in *CNR2*, and rs1800437 in *GIPR* could benefit from both antiresorptive and osteoanabolic treatment; and patient #14 with rs1042138 in *CALCR* may benefit from antiresorptive drug prescriptions.

## Figures and Tables

**Table 1:** Information about patients.

Patient	Age	Sex	Hypertension Grade	Cardiovascular Disease Risk (CVD Risk)	Chronic Gastritis	Diabetes Mellitus	Smoker/Nonsmoker Status	Other
#7	69	F	Grade 3	CVD 4	+	-	Unknown	
#9	51	F	-	-	+	-		
#10	71	F	Grade 2	CVD 3	+	-		
#14	73	M	Grade 2	CVD 4	+	Type 2		Angina pectoris
#15	64	M	-	-	+	-		
#21	70	F	No information	No information	No information	No information		
#8 (control)	60	F	Grade 2	CVD 2	+	-		BMI 35–40 (class 2 obesity)

## Data Availability

All data can be obtained from the corresponding author upon reasonable request.

## Supplementary Materials

The following supporting information can be downloaded at: https://www.mdpi.com/article/10.3390/ijms252413594/s1.
