# Supplementary material for: SNPs in GPCR Genes and Impaired Osteogenic Potency in Osteoporotic Patient Lines-Based Study
Source: Int J Mol Sci. 2024 Dec 19;25(24):13594. doi: 10.3390/ijms252413594 (PMC11677449; doi:10.3390/ijms252413594)
Supplement: Supplementary file 1 [file ijms-25-13594-s001.zip › ijms-3364060-SI.pdf]

Table S1. Oligonucleotides used in this work

Oligonucleotide	5'-3' sequence
RUNX2 F	GAGTGGACGAGGCAAGAGT
RUNX2 R	GGGTTCCCGAGGTCCATCTA
COL1A1 F	GACCTAAAGGTGCTGCTGGAG
COL1A1 R	CTTGTTCACCTCTCTCGCCA
OGN F	GGCAATAACACCATTACCTCCC
OGN R	AGGGTGGTACAGCATCAATGT
POSTN F:	CCCAGCAGTTTTGCCCATT
POSTN R	TGTGGTGCTCCCACGAT
BGLAP F	GGCAGCGAGGTAGTGAAGAG
BGLAP R	CTGGAGAGGAGCAGAACTGG
GAPDH F	AATGAAGGGGTCATTGATGG
GAPDH R	AAGGTGAAGGTCGGAGTCAA.
